# Random forest machine learning method outperforms prehospital National Early Warning Score for predicting one-day mortality: A retrospective study

**DOI:** 10.1016/j.resplu.2020.100046

**Published:** 2020-12-05

**Authors:** Jussi Pirneskoski, Joonas Tamminen, Antti Kallonen, Jouni Nurmi, Markku Kuisma, Klaus T. Olkkola, Sanna Hoppu

**Affiliations:** a Department of Emergency Medicine and Services, University of Helsinki and HUS Helsinki University Hospital, Helsinki, Finland; b Faculty of Medicine and Health Technology, Tampere University, Tampere, Finland; c Emergency Medical Services, Tampere University Hospital, Tampere, Finland; d Department of Anaesthesiology, Intensive Care and Pain Medicine, University of Helsinki and HUS Helsinki University Hospital, Helsinki, Finland

**Keywords:** Emergency medical services, Prehospital, Cardiac arrest prevention, Early warning score, National Early Warning Score, NEWS, Random forest, Machine learning

## Abstract

**Aim of the study:**

The National Early Warning Score (NEWS) is a validated method for predicting clinical deterioration in hospital wards, but its performance in prehospital settings remains controversial. Modern machine learning models may outperform traditional statistical analyses for predicting short-term mortality. Thus, we aimed to compare the mortality prediction accuracy of NEWS and random forest machine learning using prehospital vital signs.

**Methods:**

In this retrospective study, all electronic ambulance mission reports between 2008 and 2015 in a single EMS system were collected. Adult patients (≥ 18 years) were included in the analysis. Random forest models with and without blood glucose were compared to the traditional NEWS for predicting one-day mortality. A ten-fold cross-validation method was applied to train and validate the random forest models.

**Results:**

A total of 26,458 patients were included in the study of whom 278 (1.0%) died within one day of ambulance mission. The area under the receiver operating characteristic curve for one-day mortality was 0.836 (95% CI, 0.810−0.860) for NEWS, 0.858 (95% CI, 0.832−0.883) for a random forest trained with NEWS variables only and 0.868 (0.843−0.892) for a random forest trained with NEWS variables and blood glucose.

**Conclusion:**

A random forest algorithm trained with NEWS variables was superior to traditional NEWS for predicting one-day mortality in adult prehospital patients, although the risk of selection bias must be acknowledged. The inclusion of blood glucose in the model further improved its predictive performance.

## Introduction

The National Early Warning Score (NEWS) is a validated method for predicting deterioration in hospital wards.^1^, ^2^ It has been shown to predict short-term mortality in prehospital environments in retrospective studies,^3^, ^4^, ^5^, ^6^, ^7^ but its role in prehospital clinical decision making remains controversial.^8^

Recent in-hospital studies have demonstrated that novel machine learning methods can surpass traditional early warning scores in predicting admission, the need for intensive care and short-term mortality at emergency departments as well as in detecting impending sepsis in wards.^9^, ^10^, ^11^, ^12^ However, information from prehospital environments is scarce.^13^ Such machine learning methods could be trained to consider a number of the prognostically valuable variables that are recorded in prehospital electronic patient record systems. For instance, it has been suggested that adding blood glucose as a physiological parameter into the NEWS system could improve its predictive performance.^14^

In this study, we aimed to compare the predictive performance of NEWS and random forest machine learning models incorporating NEWS variables and blood glucose for one-day mortality in previously collected prehospital material.

## Methods

### Ethical considerations

The study protocol followed the principles of the Declaration of Helsinki and was approved by the Department of Emergency Medicine and Services, HUS Helsinki University Hospital (§68, 11.11.2015). No informed consent or ethics committee approval is required by Finnish legislation for a retrospective registry study such as this.

### Study population

We collected all of the electronic ambulance mission reports in the Helsinki and Uusimaa Hospital District, Finland, made between August 17th 2008 and December 18th 2015, excluding cases without the vital signs required to calculate NEWS values and blood glucose measurement. By excluding all patients with these missing variables, we maximised the quality of the data for statistical analysis and avoided imputations in machine learning model training, while recognising the possibility of causing selection bias. In a secondary analysis, cases with appropriate data to calculate NEWS but possibly unknown blood glucose measurement were examined. Study area EMS system and dispatch process are described in detail elsewhere.^6^

### Data handling and statistical analysis

The mission data had been recorded in an electronic patient record system (Merlot Medi, CGI Suomi Oy, Helsinki, Finland). The physiological variables of oxygen saturation, heart rate and blood pressure were automatically recorded from monitors whereas respiratory rate, body temperature, level of consciousness and oxygen use required manual input. The initial values for each physiological variable were used for the analysis, except for heart rate and oxygen saturation for which a mean of the first five minutes was used. One-day mortality was selected as our primary outcome since it was considered to be suitable for prehospital setting regarding clinical decision making.^3^ The Digital and Population Data Services Agency.

As this was post-hoc analysis, no power calculations were performed for this specific research question. Statistical analysis was performed using Python (version 3.6.9), and the main statistical packages used were NumPy (version 1.17.3) and sklearn (version 0.21.3).

We selected the random forest as the machine learning method for this study as it has been shown to outperform traditional regression.^15^ It is a supervised machine learning approach known to extract information from noisy input data and learn highly nonlinear relationships between input and target variables. Random forest models are very resistant to overfitting and can learn from imbalanced predictor class presentation.

Random forests are a collection of computer-generated decision trees. A single decision tree is not able to on complex problems, but a collection of these weak learners has been shown to work well in many prediction tasks involving human physiology.^16^ In order to train a random forest, a training feature space is randomly populated with a uniform sampling of input feature thresholds at each split node in order to maximise information gain for the entire forest.^17^

Model evaluation was performed using ten-fold stratified cross-validation in which training is followed by testing for ten times. Each fold presents an independent data subset to the random forest algorithm and uses a different data subset to estimate predictive performance using the area under the receiver operating characteristic curve (AUROC) performance metric. These generated folds were later used to computationally estimate confidence intervals for the different predictors using bootstrap resampling with 10,000 sample points as the normality of cross-validated AUROC scores is not guaranteed. The overall performance of the model is the combination of the bootstrap samples from the ten testing folds (i.e. AUROC distributions). 95% confidence intervals (CI) were calculated for the continuous variables; all AUROC results are presented with 95% CI in parentheses. Bootstrapping method was also used to estimate p-values (null hypothesis for equal AUROCs) numerically.

## Results

**Fig. 1:**
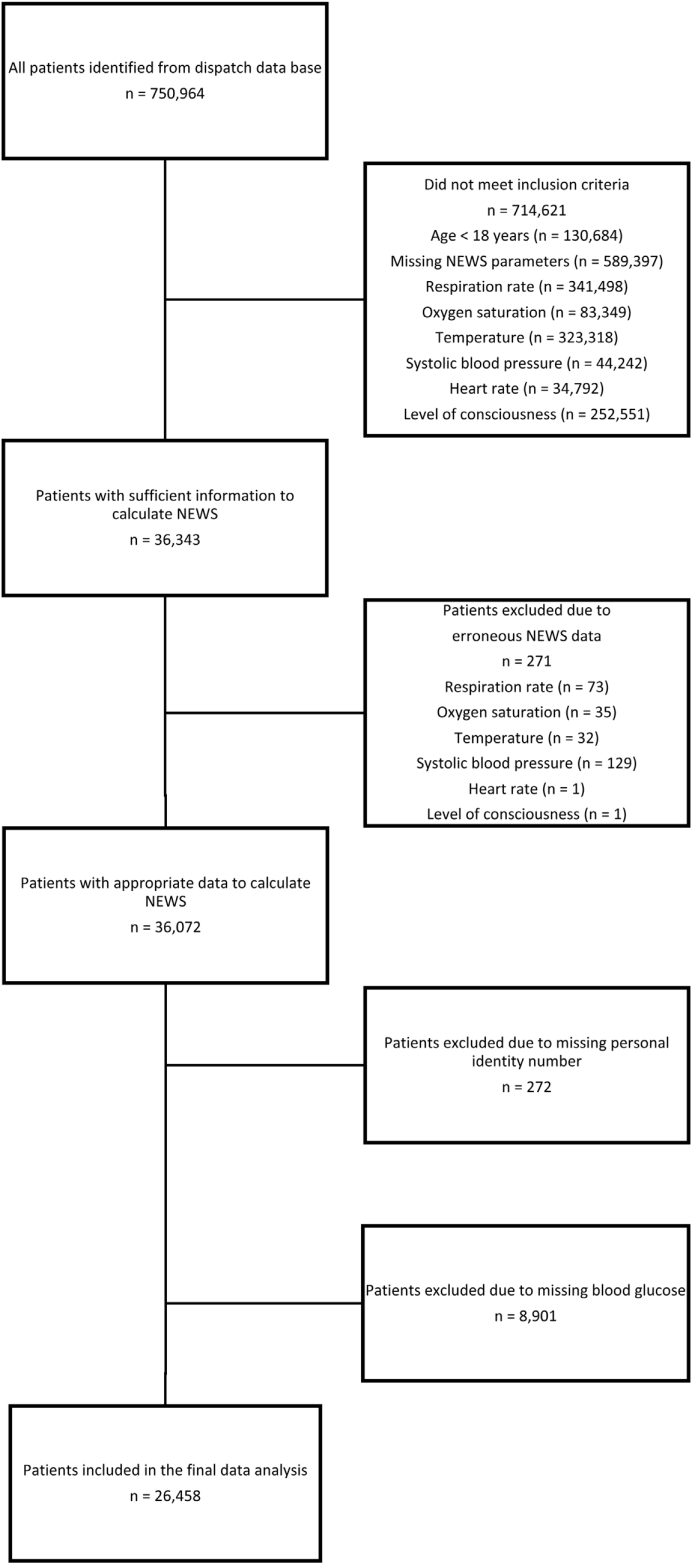
Flow chart of study cohort selection.

A total of 26,458 prehospital EMS patients were included in the study (Fig. 1). Of these patients, 278 (1.0%) died within one day. None of the deaths occurred at the scene. The demographic characteristics of included and excluded patients are presented in Table 1. Prehospital use of supplemental oxygen was more common in the study cohort patients, but otherwise the groups were similar in terms of NEWS variables.

**Table 1:** Characteristics of the study cohort and overall adult population.

	Study cohort	All patients age > 18 years
n	26,458	620,280
Age, mean, SD (years)	65.6, 19.9	60.6, 21.4
Male sex, n, %	12,783, 48.3%	n/a
NEWS, median, IQR	3, 1–6	n/a
Respiration rate, median, IQR (min^−1^)	16, 15–20	16, 15 – 18
Blood oxygen saturation, median, IQR (%)	96, 93–98	97, 95 – 98
Use of supplemental oxygen, n, %	4,564, 17.2%	41,669, 6.7%
Body temperature, median, IQR (ºC)	36.8, 36.3–37.3	36.8, 36.4–37.3
Systolic blood pressure, median, IQR (mmHg)	142, 123–164	141, 124–160
Heart rate, median, IQR (min^−1^)	87, 73–103	86, 74–101
Level of consciousness on AVPU scale, n, %		
Alert	20,281, 76.6%	n/a
Reacts to voice	2,507, 9.5%	n/a
Reacts to pain	2,246, 8.5%	n/a
Unresponsive	1,424, 5.4%	n/a
Blood glucose, median, IQR (mmol/l)	7.2, 6.0–9.1	n/a
Primary complaint, n, %		
Trauma	1,757, 6.6%	130,538, 21.0%
Medical	24,701, 93.4%	489,742, 79.0%

IQR: interquartile range, n/a: not available.

**Fig. 2:**
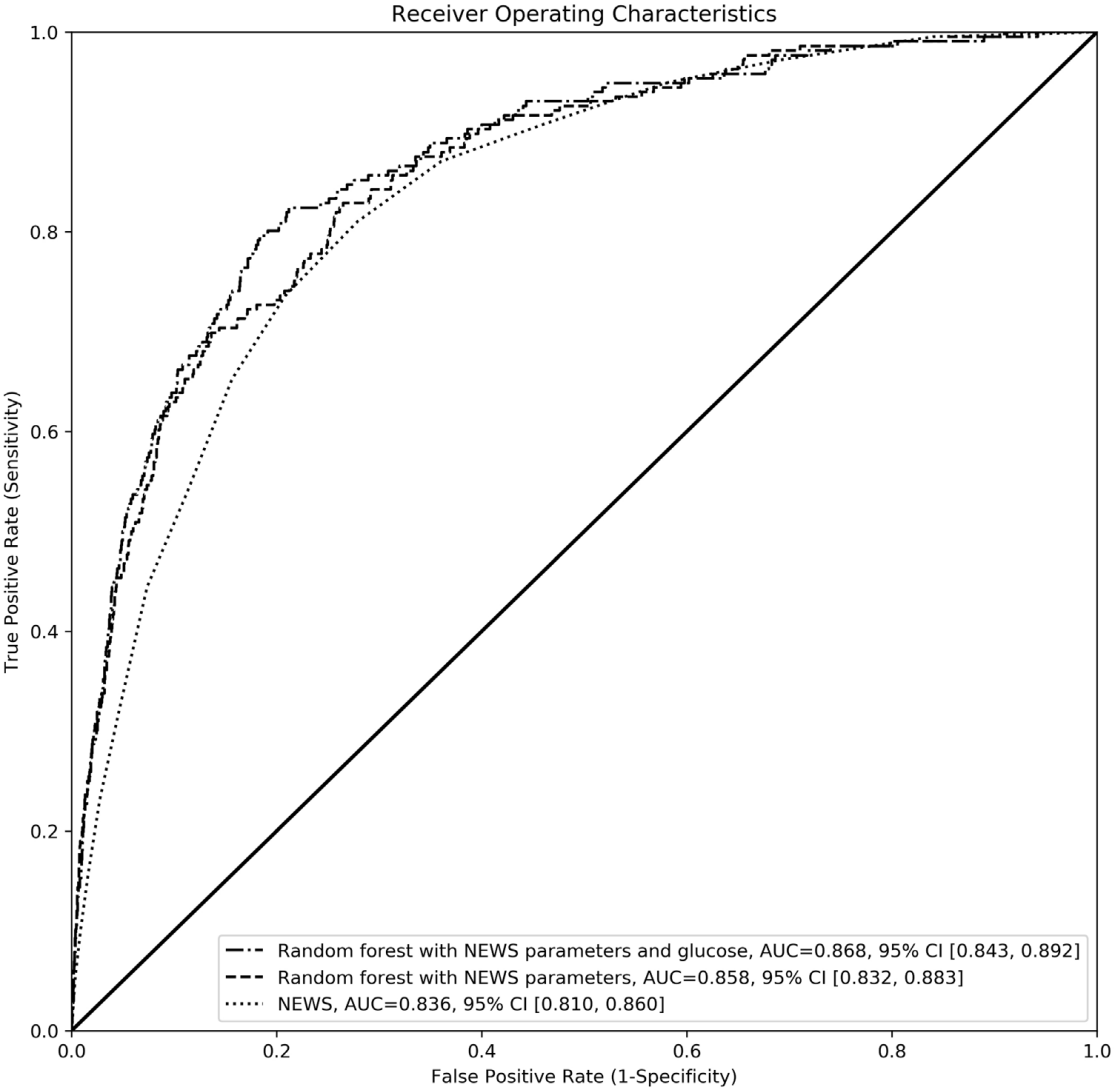
Receiving operating characteristics curves for the three models: model based on NEWS score, model based on random forest trained with NEWS variables data and model based on random forest trained with NEWS variables data and blood glucose. Random forest produces a prediction as a probability and NEWS scores may be also interpreted as a probability when scaled with the maximum score value.

The AUROC for one-day mortality using NEWS was 0.836 (95% CI 0.810−0.860). The corresponding AUROC values determined with the random forest models trained with NEWS variables only and with NEWS variables and blood glucose were 0.858 (0.832−0.883) and 0.868 (0.843−0.892), respectively (Fig. 2). The AUROC of the random forest models were significantly higher than that of NEWS (P = 0.005 NEWS variables only and P 0.001 NEWS variables and glucose). The AUROCs of the two random forests also differed significantly (P = 0.032).

**Fig. 3:**
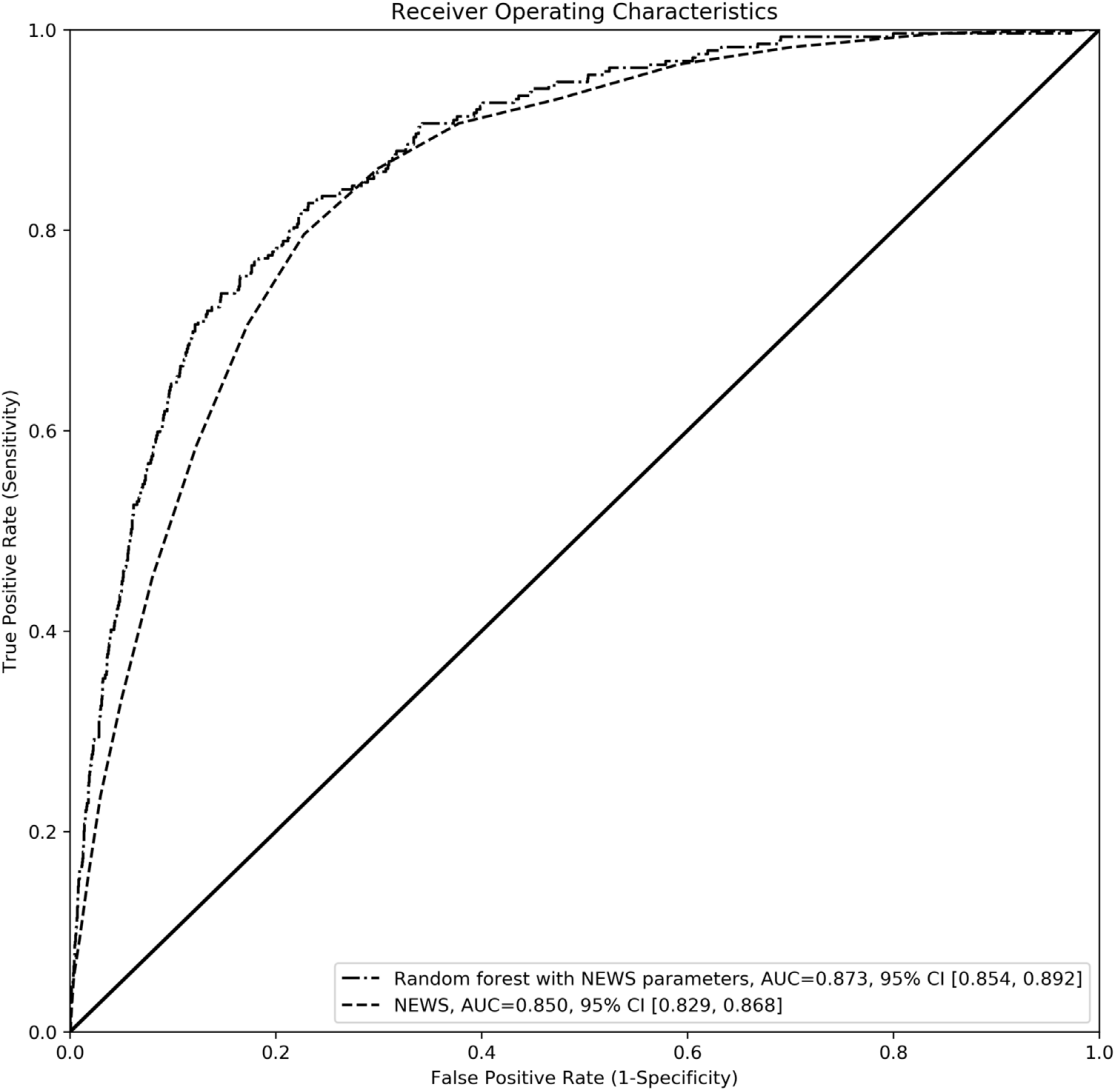
Receiving operating characteristics curves for a model based on NEWS score and a model based on random forest trained with NEWS variables.

In a secondary analysis regarding patients with all NEWS variables measured (n = 35,800), the AUROC for one-day mortality using NEWS and random forest trained with NEWS variables only were 0.850 (95% CI 0.829–0.868) and 0.873 (95% CI 0.854–0.892, P < 0.001 compared with NEWS), respectively (Fig. 3).

## Discussion

### Principal findings

In the present study, a random forest machine learning method using NEWS variables outperformed NEWS in predicting one-day mortality in adult prehospital patients, both with and without a blood glucose variable.

### Relation of results to other studies

Our results support the recent results of Spangler et al.^13^ and confirm their finding of a machine learning method surpassing a traditional NEWS approach to prehospital risk stratification. Although a different machine learning method was used and a composite risk score of multiple outcomes was assessed, their data nevertheless further demonstrate the feasibility of using machine learning approaches to prehospital risk assessment. Our results are also in line with in-hospital emergency department studies that have compared machine learning to traditional early warning scores or triage tools and shown improved predictive performance by the machine learning techniques.^9^, ^10^, ^11^

### Relevance of the study results

NEWS may not be the optimal tool for detecting impending cardiac arrest in prehospital settings since randomly selected prehospital patients may differ in terms of factors predicting mortality from in-hospital ward patients for whom NEWS was originally developed. As such, the physiological thresholds that are used in NEWS may not be valid. In a systematic review regarding the performance of prehospital NEWS, the authors concluded that only extreme aggregate scores (i.e. NEWS = 0 or 7) could reliably predict clinically relevant outcome.^8^ Use of the random forest method allows for more precise physiological weighting and can model complex nonlinearities in a given population. It also allows the incorporation of multiple variables including factors beyond the traditional vital signs, such as blood glucose which has been shown to improve mortality prediction in this context.^14^

On the other hand, on a secondary analysis performed on a larger cohort of patients focusing solely on NEWS parameters without glucose, the performance slightly improved, although the statistical significance of this improvement could not be tested due to the differing patient cohorts. We speculated this was likely due to the larger data set including a higher number of mortalities and therefore presenting more learning targets for random forest. This outlines the rationale of NEWS in including relevant physiological parameters to predict short-term mortality, which can still be utilized for even better predictions when analysed using novel methodology such as random forest.

The purpose of all early warning scores is to assist the detection of physiological abnormalities before they lead to cardiac arrest, and so a machine learning model could help reduce by improving the detection of patients at risk.^18^ Training the model with local data would help overcome issues of generalisability of data from other populations. In that way, the model would adapt to the system-specific population and could be retrained over time with larger datasets or respond to changes in care guidelines or population demographics. In EMS that operate with electronic patient record systems, introducing automatically computed predictions for short-term mortality at the scene could help in patient-specific decision making when personnel need to consider the urgency of transport or non-conveyance. At this moment, these predictions may provide some guidance to clinicians as they are not standalone risk stratifications systems yet.

### Strengths and limitations of the study

This was a retrospective study and the results are not fully generalisable because of selection bias risk from the very large exclusion rate and the fact that some mission data are collected over ten years ago. Missing values were not imputed, which is an important limitation of the study. Despite this, and the lack of power calculations, we consider the cohort of 26,489 patients including 278 mortalities sufficiently powered. Decisions to limit care such as do not resuscitate orders are not systematically entered in prehospital patient records, and it is possible that the existence of such orders could have affected the outcome of some patients.

All machine learning methods have common inherent limitations and ‘artificial intelligence’ should be considered as a sophisticated algorithm which can give accurate answers to a simple and narrow question. We are aware that our random forest model is a more complicated version but a more powerful version of NEWS which is tailored in our system. The most significant limitation of the random forest approach is the non-generalisability of the dataset since the exact AUROC value is likely to differ across different patient cohorts. However, we compared the same dataset using different predictive models in this study and therefore believe that the observed differences in the predictive values for one-day mortality of prehospital patients are true. Another important limitation concerning random forest is that it has a ‘black box’ element. As the name of the method implies, hundreds of decision trees are randomly generated into the model. Their clinical interpretation is extremely difficult although the decisions trees can be visualised (Supplemental Figure 4).

### Future studies

A further prospective study is warranted to validate this new risk stratification model. Taking into account the issues of generalisability, a similar study in a different prehospital population could be considered. Given that a small minority of patients involved in EMS missions die within one day of contact, other outcomes such as the use of emergency department resources or the need for hospitalisation should also be looked at in future studies. In addition, further research into other possible variables to be considered in machine learning models is essential.

## Conclusions

We have demonstrated that a random forest machine learning model was superior to NEWS in predicting one-day mortality in adult prehospital patients.

## Conflicts of interest

None.
