# Supplementary material for: Random forest machine learning method outperforms prehospital National Early Warning Score for predicting one-day mortality: A retrospective study
Source: Resusc Plus. 2020 Dec 5;4:100046. doi: 10.1016/j.resplu.2020.100046 (PMC8244434; doi:10.1016/j.resplu.2020.100046)
Supplement: Supplementary file 1 [file mmc1.docx]

Supplementary Appendix for the paper **Random forest machine learning method outperforms prehospital National Early Warning Score for predicting one-day mortality: a retrospective study**

By Jussi Pirneskoski et al.

Supplemental Figure 1


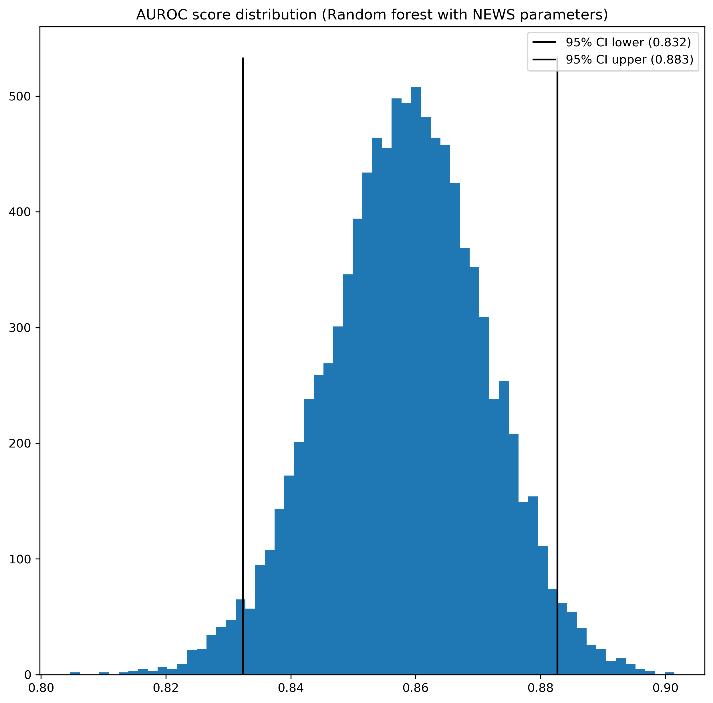

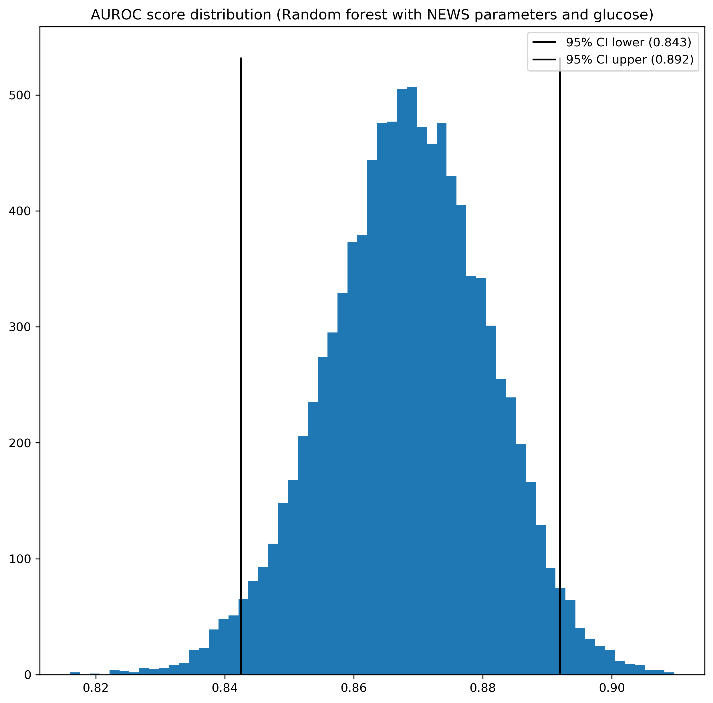

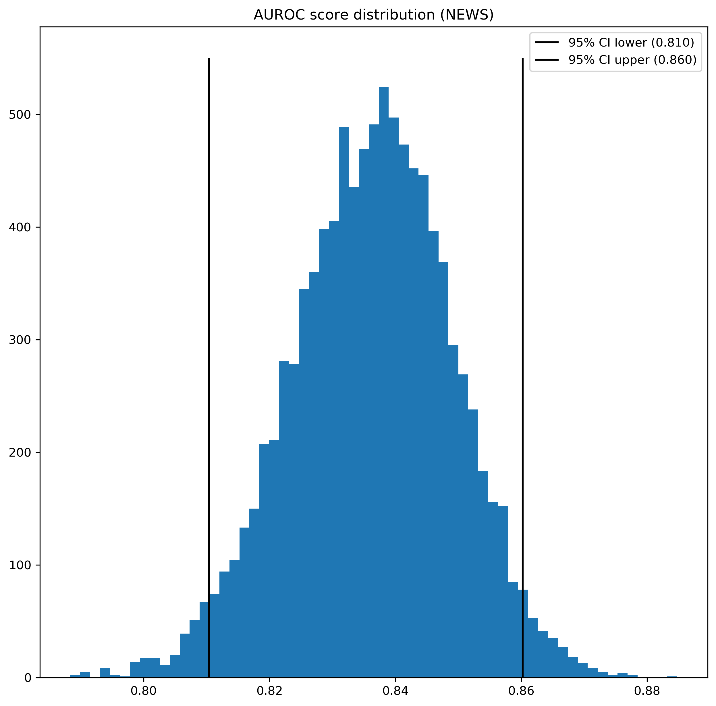


Numerical calculation of 95% confidence intervals for AUROC from the bootstrapped AUROC distribution.

Supplemental Figure 2


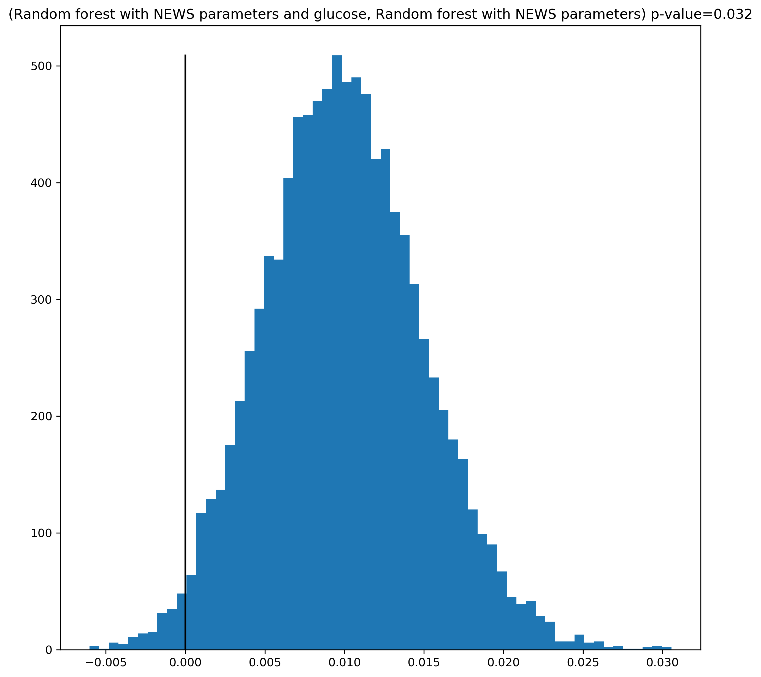

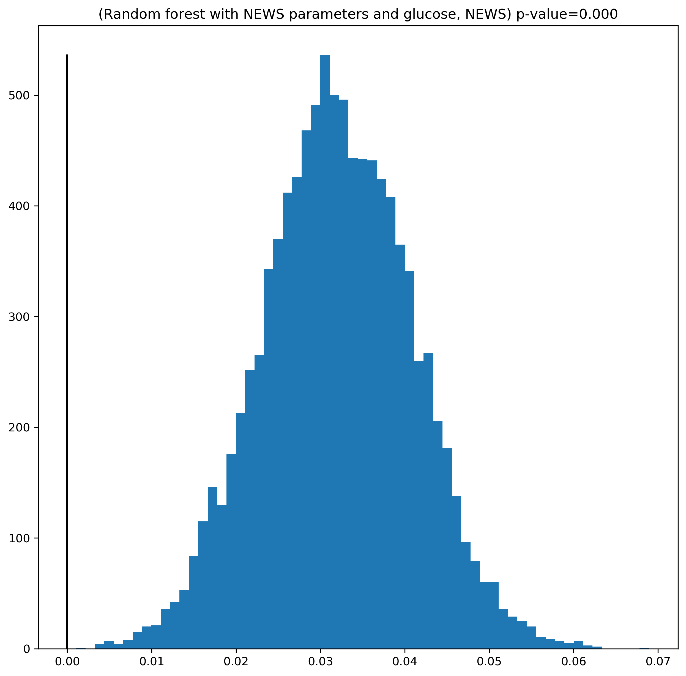


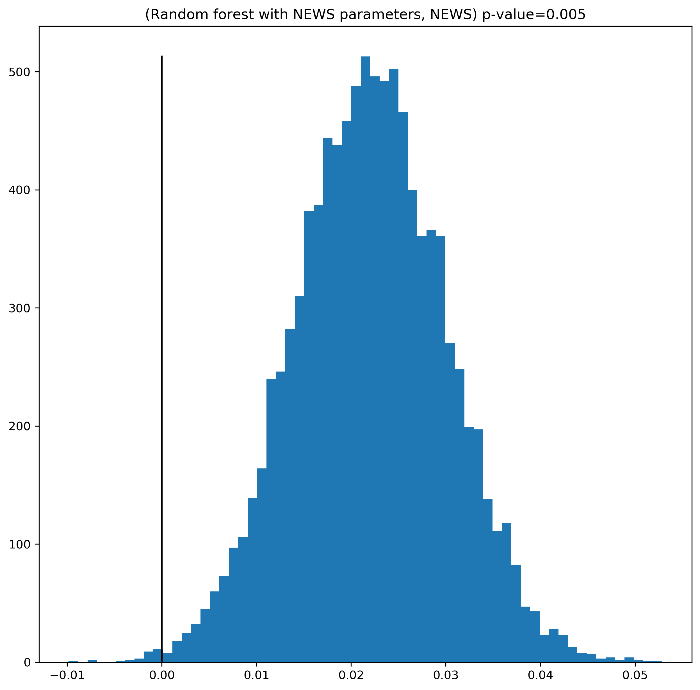


Numerical calculation of p-values from bootstrapped Z-score distribution.

Supplemental Figure 3.


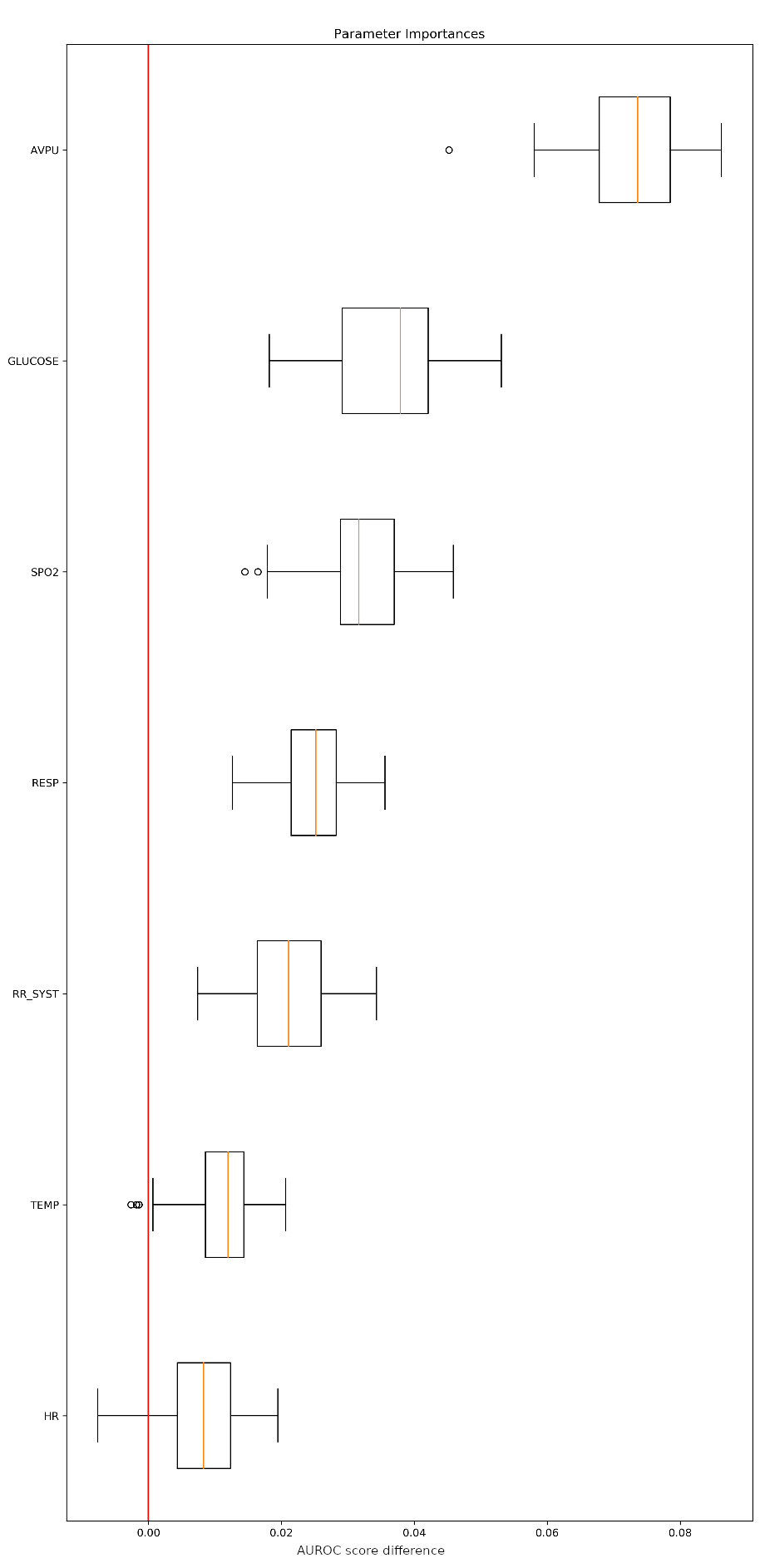


Permutation importances for each predictor in the random forest model trained with NEWS variables and blood glucose. Positive difference from zero baseline indicates higher variable importance.

Supplemental Figure 4.


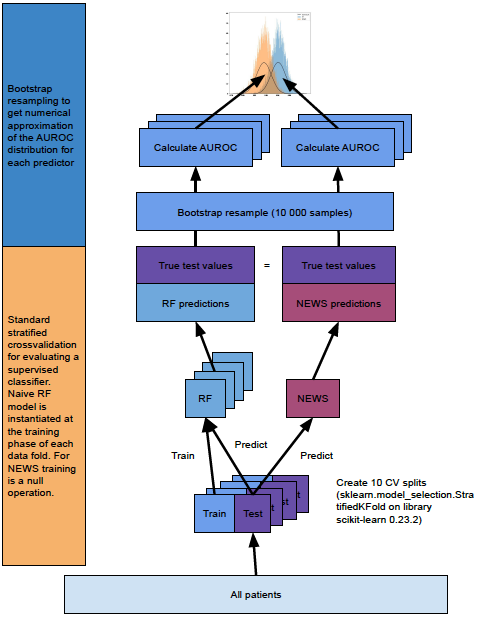


Flow chart depicting the process of data analysis. RF: random forest; CV: cross-validation

Supplemental Figure 5.


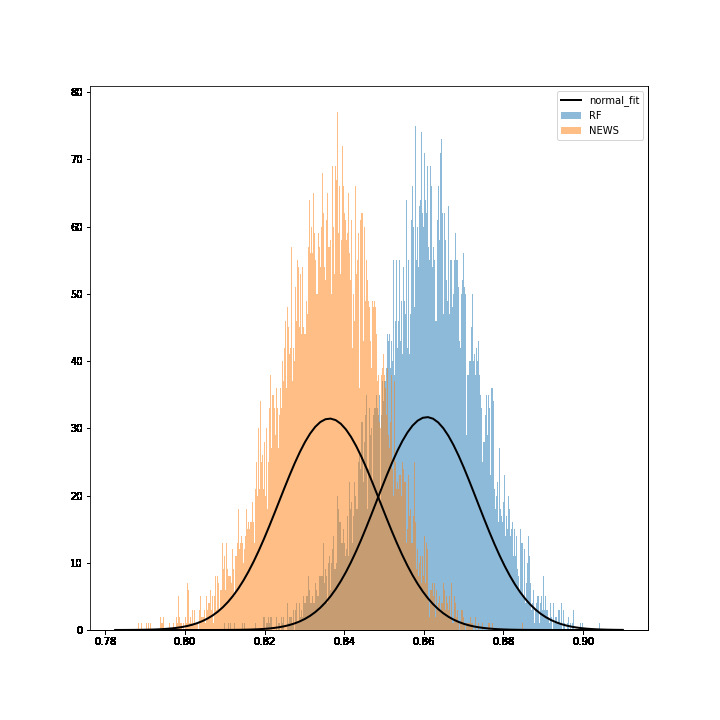


Visualization of the bootstrapped AUROC distributions for NEWS and random forest models.

Supplemental Figure 6.

A B


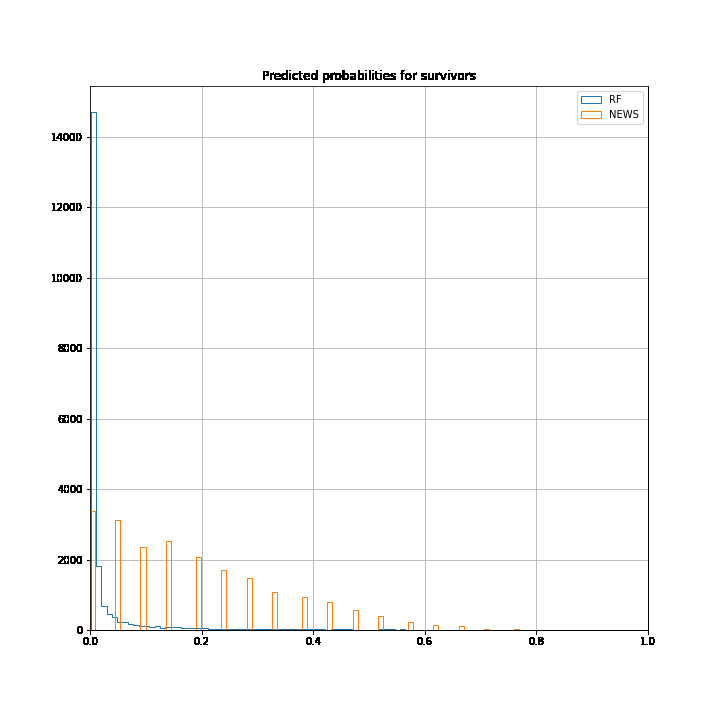

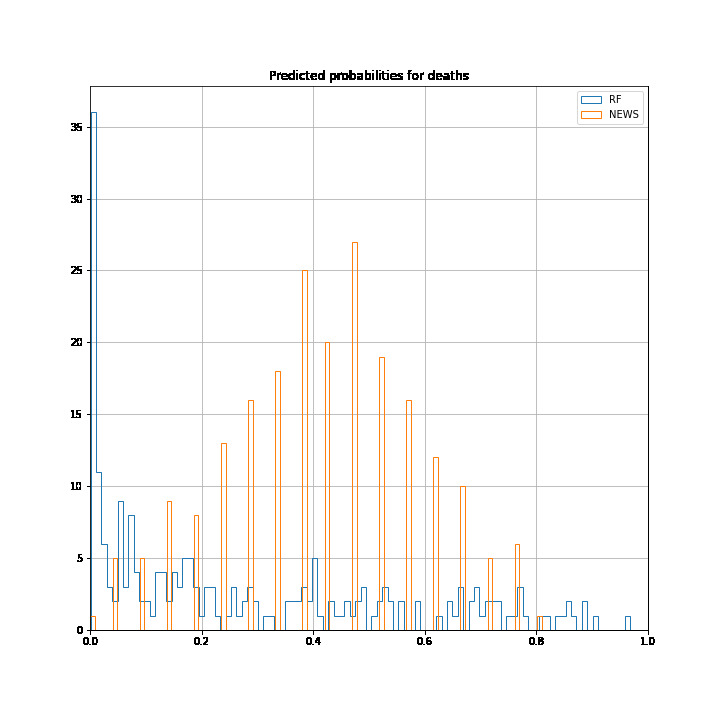


Comparison of predicted probabilities for survivors (Panel A) and non-survivors (Panel B) between NEWS model and random forest model. Random forest model is more sensitive at low false positive rates than NEWS.
